# Object speed and distractor number affect performance but not attentional allocation in multiple object tracking

**DOI:** 10.3758/s13414-026-03343-3

**Published:** 2026-10-12

**Authors:** N. Adamian, F. Akalan, S. K.Andersen

**Affiliations:** 1 Research Centre for Brain and Behaviour, Liverpool John Moores University, Tom Reilly Building, Byrom Street, Liverpool, L3 3AF UK; 2 University of Aberdeen, Aberdeen, UK; 3 University of Southern Denmark, Odense, Denmark

**Keywords:** SSVEP, EEG, Multiple object tracking, Spatial attention, Selective attention

## Abstract

**Abstract:**

Keeping track of multiple moving objects across dynamic real-world scenarios such as driving, team sports, or crowded social environments is a fundamental challenge for visual attention. We have previously demonstrated that as the number of tracked objects increases, the strength of attentional facilitation allocated to each individual object decreases, limiting tracking success. It is also well established that beyond the number of tracked objects, faster-moving objects and objects embedded amongst higher numbers of distractors are more difficult to track. Are these effects on tracking difficulty also mediated by less effective allocation of attention to tracked targets as in the case of tracking more targets? If so, one should expect the strength of attentional modulation to drop systematically with increasing speed and total number of moving stimuli. In two experiments (total *n* = 70), participants were instructed to track moving targets amongst identical distractors while we manipulated object speed (Experiment 1) and number (Experiment 2). As expected, tracking performance declined with both manipulations. However, steady-state visual evoked potentials (SSVEPs) recorded during successful tracking revealed that attentional enhancement of tracked targets compared with distractors did not drop with increasing speed or object number. In summary, bottom-up changes in the stimulus display and top-down attentional manipulations affect tracking performance in independent ways, with the balance between strength of attentional allocation and bottom-up demands of the task determining successful tracking. The allocation of attention itself seems to be determined exclusively by top-down goals rather than being reactive to bottom-up display characteristics.

**Significance statement:**

Keeping track of multiple moving objects is fundamental to navigating dynamic real-world scenarios. This ability is accomplished through multifocal attentional selection, which weakens as the number of tracked targets increases. This study asks whether other stimulus manipulations increase tracking difficulty by diluting attentional allocation. Using steady-state visual evoked potentials to measure selective attention during tracking, we demonstrate that both increases in speed and distractor number impair performance, but they do not affect attentional enhancement of targets relative to distractors. This suggests that top-down attentional control operates independently from bottom-up demands.

## Introduction

Human observers demonstrate a remarkable ability to visually track multiple, independently moving objects, even when these targets are embedded amongst identical distractors (Holcombe, 2023; Pylyshyn & Storm, 1988). The mechanisms enabling us to simultaneously track multiple moving objects, and the fundamental limitations on this capacity, have been a central focus of research for decades (Meyerhoff et al., 2017).

The limits on tracking manifest across different aspects of the dynamic display. One of the most fundamental constraints is the limit on the number of objects that can be continuously tracked. While early research suggested that capacity was fixed at four objects (Pylyshyn & Storm, 1988), this theory was at odds with the discovery of a smooth trade-off between the number of tracked objects and their speed. When objects move slowly, participants can track up to as many as eight to nine objects, whereas at high speeds they may only be able to track a single one (Alvarez & Franconeri, 2007; Holcombe & Chen, 2012; Liu et al., 2005; Tombu & Seiffert, 2011). This finding was interpreted as evidence of a limited resource that is flexibly divided among the targets in line with task demands. The more difficult tracking gets, for example due to increased proximity of objects in the display, the more “resource” each target needs in order to be successfully tracked, which in turn limits tracking capacity (Iordanescu et al., 2009).

Numerous studies confirmed that tracking performance declines as object speed increases (Bettencourt & Somers, 2009; Drew et al., 2013; Feria, 2013; Tombu & Seiffert, 2011), although the underlying mechanisms remain debated. One possibility, in line with the aforementioned flexible resource model (Alvarez & Franconeri, 2007), is that faster moving objects increase attentional demands on tracking, and thus speed itself affects tracking performance (Feria, 2013; Holcombe & Chen, 2012; Tombu & Seiffert, 2011). Alternatively, it has been proposed that increased speed reduces tracking performance solely because it increases the number of close encounters between objects, leading to spatial interference and target–distractor confusions (Franconeri et al., 2008, 2010).

Another confirmed prediction of the spatial interference account of multiple object tracking is that for a fixed number of targets, tracking becomes more difficult when additional distractors are present (Bettencourt & Somers, 2009; Drew et al., 2013; Feria, 2012). This increases the likelihood of targets and distractors coming in close proximity and causing interference, which can be overcome through increasing attentional demands on tracking (Franconeri et al., 2010). However, Bettencourt and Somers (2009) proposed a different explanation—if distractors require active suppression, they take away from the attention available for targets, and thus tracking becomes more difficult with more distractors.

Overall, while there is consensus that tracking is flexible, capacity-limited, and relies on attention, the nature of this attentional resource and the ways in which flexibility manifests remain debated. A recent study from our lab (Adamian & Andersen, 2022) demonstrated that the magnitude of attentional amplification of tracked targets relative to distractors measured with steady-state visual evoked potentials (SSVEPs) decreased with increasing set size. Moreover, this strength of attentional selection was predictive of tracking success on a given trial. This suggests that the allocation of attention in early visual cortex limits how many objects can be simultaneously tracked. The present study extends this work by examining whether attentional amplification of targets depends on other factors known to affect tracking: object speed and the total number of objects present.

In two experiments, participants tracked moving objects among identical distractors. Targets and distractors flickered at different frequencies, each eliciting steady-state visual evoked potentials (SSVEPs) at their respective flicker frequencies. This frequency-tagging approach resulted in separable neural activity for each stimulus type (Norcia et al., 2015). SSVEPs directly index visual stimulus processing and are highly sensitive to changes in physical properties such as size (Chen et al., 2019), contrast (Di Russo et al., 2001; Hedjar et al., 2025; Kaneko et al., 2020), or eccentricity (Walter et al., 2012). SSVEPs are also highly sensitive to the modulation of visual processing by selective attention (Adamian & Andersen, 2024; Morgan et al., 1996). Prior studies combining manipulations of covert selective attention and physical stimulus properties, such as contrast (Andersen et al., 2012), stimulus eccentricity (Walter et al., 2012), or combination of chromatic and luminance signals (Martinovic et al., 2018), have found that such physical manipulations produce larger effects on SSVEPs than selective attention. In the present experiments, this allowed us to concurrently measure the effects of physical and attentional manipulations and their interactions on visual processing of both targets and distractors throughout the tracking period.

In Experiment 1, the speed of motion was manipulated while the number of targets and distractors was constant. In Experiment 2, the speed was constant as was the number of targets (four); however, the number of distractors was manipulated. In both experiments, the key variable of interest is the difference in SSVEP amplitudes elicited by targets and distractors. If object speed or total number of objects affects how limited attention is distributed—for example, if faster speeds result in attention not ‘keeping up’ with motion or if extra items challenge the spatial resolution of attention—we would expect a decline in attentional selectivity (reflected in a smaller difference in SSVEP amplitudes between targets and distractors) corresponding to decline in tracking performance.

## Experiment 1: Speed

### Method

Data collection and analysis of Experiment 1 were preregistered (https://osf.io/pxh25).

#### Participants

A target sample size of 17 participants was determined through power analysis (detailed in the preregistration protocol). Twenty-two members of the University of Aberdeen student community participated in the study (six men, all right-handed; *M*_age_ = 26 years, *SD*_age_ = 6.08). All participants provided written informed consent and received £20 for their time. Participants reported normal colour vision and normal or corrected-to-normal visual acuity. The study received ethical approval from the Psychology Ethics Committee at the University of Aberdeen (PEC/5048/2022/7).

Data from five participants were excluded because fewer than 20 epochs remained in at least one experimental condition after rejecting trials with EEG artefacts and trials with incorrect behavioural responses, resulting in a final sample of 17 participants.

#### Stimuli and procedure

Stimuli were created using MATLAB (MathWorks Inc., Natick, MA) and Psychtoolbox (Kleiner et al., 2007). They were presented in a dimly lit room on a 32-in. Display++ monitor (CRS, UK) with a resolution of 1,920 × 1,080 and refresh rate of 120 Hz. Participants were seated at a viewing distance of approximately 60 cm without head restraint and instructed to maintain fixation on a central fixation point (0.3 dva).

Stimuli were presented against a mid-grey background (29 cd/m^2^) within a centrally positioned light-grey elliptical field (38.5 cd/m^2^, 36 dva width, 28 dva height). The display consisted of eight identical red discs (12.2 cd/m^2^, 4 dva diameter). Target cues were provided by black outlines around each disc, whilst feedback was given by outlining cued discs in green (correct) or red (incorrect).

**Fig. 1:**
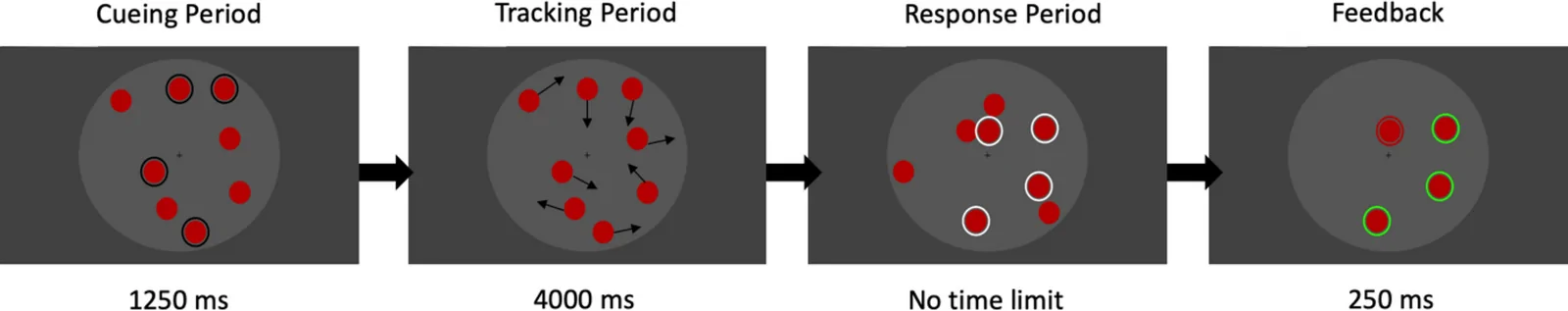
Illustration of the trial sequence in Experiment 1. Targets and distractors flickered at designated frequencies throughout cueing and tracking phases. Targets and distractors moved at one of three speeds during the tracking phase. Black arrows indicate motion directions and were not displayed. Participants responded using consecutive mouse clicks and could select or deselect objects as needed. They could only proceed to the next trial once exactly four objects were selected. (Colour figure online)

Each trial began with the eight red discs randomly positioned within the elliptical viewing area (see Fig. 1). Four discs were then outlined as to-be-tracked targets. After 1,250 ms, the outlines disappeared and all objects began moving linearly in randomly chosen directions, bouncing off each other and the borders of the background field at physically realistic angles. Throughout both cueing and tracking, all objects flickered at their designated frequencies. To prevent discs from overlapping, they were surrounded by an invisible “bumper” border 1 dva wider than the disc itself. Movement speed varied by condition: slow (3.8 dva/s), medium (7.6 dva/s), or high (10.4 dva/s). These speeds were selected to yield performance levels comparable to those observed when the number of tracked targets was manipulated in a previous study (Adamian & Andersen, 2022). After a 4,000-ms tracking period, objects stopped moving and participants selected the discs they thought were targets via mouse clicks. Participants always selected exactly four discs, guessing when necessary. Visual feedback (green outlines for correct selections, red for incorrect) and auditory feedback (high-pitch tone for all correct, low-pitch otherwise) were provided after each trial. Summary feedback was also provided after each block.

Throughout each trial, half of the discs flickered at 10 Hz whilst the other half flickered at 12 Hz. Depending on the condition, the 10-Hz discs served as targets and 12-Hz discs as distractors, or vice versa.

The experiment comprised 336 trials across eight blocks of 42 trials each, resulting in 56 trials per condition. The three speed conditions were duplicated for the two target frequencies, yielding six conditions overall. Each block of 42 trials contained exactly seven trials from each condition, which were presented in random order. A set of 56 starting positions and initial motion directions of objects were generated and copied across the six conditions (in randomized order) to minimize physical stimulus differences between conditions.

#### Behavioural data analysis

Responses in each trial were classified as correct if all four targets were reported correctly. Accuracy rates were analysed by means of a one-way repeated-measures analysis of variance (ANOVA), with the factor Speed. All ANOVA analyses used Greenhouse–Geisser correction for nonsphericity where applicable.

#### EEG acquisition and preprocessing

EEG data were recorded using an ActiveTwo amplifier system (Biosemi) from 64 Ag/AgCl electrodes at 256-Hz sampling rate. The manufacturer’s standard electrode montage was modified by relocating electrodes from T7/8 and F5/6 positions to PO9/10 and I1/2 to enhance spatial coverage over occipital regions. Electrooculographic activity was monitored using electrodes positioned above and below the left eye (vertical EOG) and at the outer canthi of both eyes (horizontal EOG). Data processing was conducted using the EEGLAB toolbox (Delorme & Makeig, 2004) and custom MATLAB (MathWorks Inc., Natick, MA) routines.

Epochs spanning 400 to 3,900 ms postmotion onset were extracted from the continuous data. Trials containing blinks or eye movements exceeding 20 μV were rejected. Each epoch was detrended by removing the epoch mean and linear trend. The preprocessed data were then submitted to an automated artefact detection routine (Junghöfer et al., 2000) that either replaces contaminated sensors using statistically weighted spherical interpolation or rejects entire trials when excessive sensors are compromised.

After removing data from participants with excessive EEG artifacts or poor behavioural performance, the average trial rejection rate was 20.1% (±9.4%), whilst the average number of interpolated channels was 3.83 (±1.24).

#### SSVEP analysis

SSVEP analysis was restricted to trials with correct behavioural responses, yielding an average of 36 epochs per condition per participant (±7.05) out of the total of 56 trials per condition. Epoched data underwent scalp current density (SCD) transformation via spherical spline interpolation (Perrin et al., 1989). Following the preregistration protocol, SSVEP amplitudes at the target frequencies (10 Hz and 12 Hz) were extracted from SCD-transformed epochs as the absolute values of complex Fourier coefficients for each frequency, condition, and participant. Prior to statistical analysis, SSVEP amplitudes for each electrode were averaged for each condition, participant and frequency and rescaled by dividing each of the resulting amplitudes by the mean over all conditions (Adamian & Andersen, 2024). The resulting amplitudes scaled to the mean of 1.0 were combined across frequencies to produce average SSVEP amplitudes for the three speed (Experiment 1) or density (Experiment 2) conditions in each cluster of electrodes. These amplitudes were submitted to a repeated measures ANOVA with the factors Attention (Attended vs Unattended) and Speed (3.8 dva/s, 7.6 dva/s and 10.9 dva/s).

#### Deviation from preregistration

Following the preregistration protocol, SSVEP amplitudes were calculated at midline occipital electrodes (Oz and POz). However, it was later decided to repeat the analysis using lateral occipital electrodes (P7/8 and PO7/8). This cluster of electrodes exhibited strong SSVEP signal, in line with a previous study using a similar paradigm (Adamian & Andersen, 2022). This study demonstrated that attentional enhancement of SSVEPs specifically in the lateral cluster of electrodes was associated with higher probability of successful tracking, suggesting particular relevance of these scalp locations for multifocal attention to moving objects. A similar lateral occipital cluster was also found to exhibit a qualitatively different integration of bottom-up (contrast) and top-down (voluntary attention) SSVEP effects in a feature-based attention task (Andersen et al., 2012), making it particularly relevant for the present investigation.

### Results

**Fig. 2:**
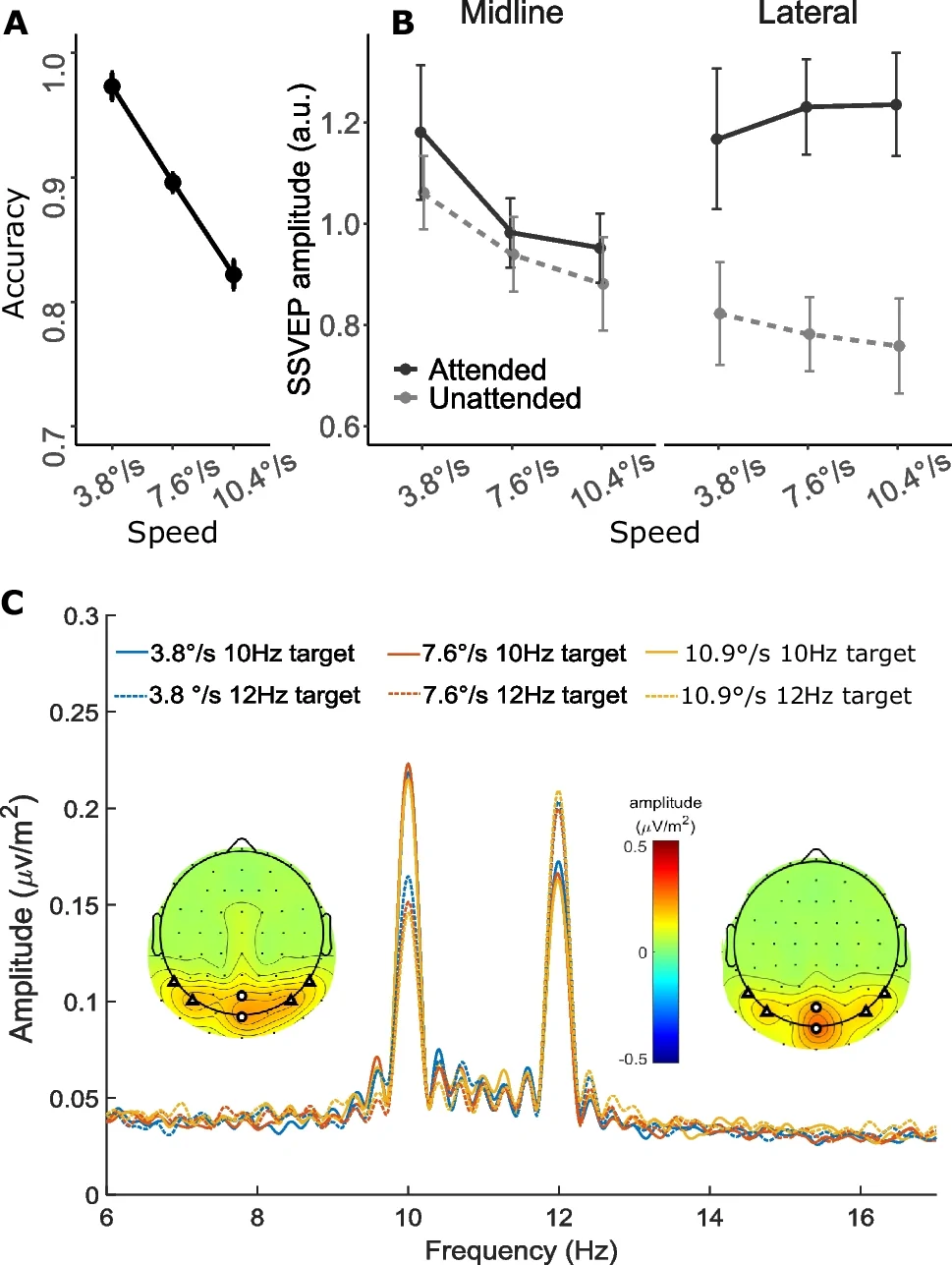
Results of Experiment 1. **A** Mean accuracy rates for three speed conditions. **B** Rescaled grand mean SSVEP amplitudes at midline and lateral electrode clusters. **C** Grand-averaged amplitude spectrum of both electrode clusters combined, obtained by Fourier transformation and zero-padded to 16,384 points. Inset: Grand mean scalp current density (SCD) maps of SSVEP amplitudes at 10 Hz (left) and 12 Hz (right) averaged across conditions. Circles on topographical maps indicate midline cluster electrodes, triangles indicate lateral cluster electrodes. Error bars denote within-subject 95% confidence intervals (Morey, 2008). (Colour figure online)

Participants made more errors when tracking faster moving objects, *F*_(2,32)_ = 37.58, *p* < 10^−8^, η^2^ = 0.35. Tracking accuracy (Fig. 2A) was reduced for the 7.6 dva/s condition (*M* = 0.90, *SD* = 0.04) compared with 3.8 dva/s (*M* = 0.97, *SD* = 0.04), *t*_(33)_ = 6.53, *p* < 10^−6^, and was lowest when tracking objects moving at 10.9 dva/s (*M* = 0.82, *SD* = 0.12), *t*_(33)_ = 6.62, *p* < 10^−6^.

#### SSVEP amplitudes

In the midline cluster of electrodes (Fig. 2B, left), SSVEP amplitudes elicited by attended objects (*M* = 1.04, *SD* = 0.12) were higher than those elicited by unattended objects (*M* = 0.96, *SD* = 0.09), *F*_(1,16) _= 5.08, *p* = 0.039, η^2^ = 0.05, BF₁₀ = 1.25, confirming that attention facilitated processing of tracked targets in early visual cortex. SSVEP amplitudes decreased with increased object speed (3.8 dva/s*: M* = 1.12, *SD* = 0.07; 7.6 dva/s: *M* = 0.96, *SD* = 0.03; 10.9 dva/s: *M* = 0.92, *SD* = 0.06), *F*_(2,32) _= 6.58, *p* =.004, η^2^ = 0.22, BF₁₀ = 1872, and there was no interaction between speed and attention, *F*_(2,32) _= 0.71, *p* =.497, η^2^ = 0.07, BF₁₀ = 0.18.

Because attentional modulation of SSVEP amplitudes is multiplicative rather than additive (Adamian & Andersen, 2024), the presence of the main effect of speed complicates direct interpretation of the interaction term. To assess whether attention effects varied across speed conditions, we therefore computed an attentional modulation index (AMI) for each condition separately, and entered AMIs into a one-way ANOVA with the factor Speed:

$$AMI= \frac{Attended-Unattended}{Attended+Unattended}$$

This additional analysis confirmed that attention was not modulated by speed (3.8 dva/s*: M* = 0.05, *SD* = 0.09; 7.6 dva/s: *M* = 0.03, *SD* = 0.06; 10.9 dva/s: *M* = 0.07, *SD* = 0.02), *F*_(2,32)_ = 0.43, *p* =.65, η^2^ = 0.01, BF₁₀ = 0.21.

In the lateral cluster of electrodes (Fig. 2B, right), SSVEP amplitudes were not modulated by object speed (3.8 dva/s*: M* = 1.01, *SD* = 0.17; 7.6 dva/s: *M* = 0.99, *SD* = 0.16; 10.9 dva/s: *M* = 0.99, *SD* = 0.22), *F*_(2,32)_ = 0.06, *p* =.946, η^2^ = 0.002, BF₁₀ = 0.09, but they were strongly enhanced by attention (Attended: *M* = 1.13, *SD* = 0.02; Unattended: *M* = 0.87, *SD* = 0.03), *F*_(1,16)_ = 52.34, *p* < 10^−6^, η^2^ = 0.38, BF₁₀ = 2.25×10^1^⁶. The interaction between speed and attention was not significant, *F*_(2,32)_ = 1.55, *p* =.228, η^2^ = 0.015, BF₁₀ = 0.28, indicating that the magnitude of attentional facilitation was stable across tracking speeds.

### Discussion

The results of Experiment 1 suggest that the strength of attentional selection of successfully tracked targets does not depend on their speed, as we observed no decrease in the relative attentional enhancement of targets compared with distractors with increased object speed in either electrode cluster. Importantly, behavioural performance was affected—participants tracked faster moving objects less successfully. The magnitude of this performance decrease is in line with the previous study where the number of tracked objects was manipulated (Adamian & Andersen, 2022). However, despite similar behavioural effects, only one of these two bottlenecks of tracking performance affects distribution of attention. While increasing the number of tracked targets weakens attentional facilitation each target receives, increasing their speed does not produce corresponding changes in attentional distribution as indexed by SSVEPs. Tracking errors due to speed therefore do not stem from weakened selection, but rather from bottom-up display characteristics that challenge this selection.

The flexible-resource model proposes that faster moving targets inherently require more attentional resources (Alvarez & Franconeri, 2007) leading to a smooth trade-off between the number of objects that can be tracked and their speed. However, it is unclear whether this trade-off results from less effective allocation of attention to faster targets or whether the same amount of attention becomes insufficient to track objects at higher speeds. In our paradigm, only speed was manipulated whilst spatial arrangement and trial duration remained constant, inevitably increasing the number of close encounters at higher speeds. Yet SSVEPs in successful tracking trials did not vary systematically with speed, suggesting that the strength of attentional selection remained constant despite increased bottom-up interference. This aligns with findings from Drew et al. (2013) who manipulated speed whilst examining tracking-related ERPs. Although their focus was on error trials, they found no effect of speed on the contralateral delay activity (CDA) when participants tracked a single target, further suggesting that speed does not modulate the attentional resources allocated to tracked objects.

Interestingly, we observed that attentional modulation was weaker in the midline electrode cluster compared with lateral clusters, and SSVEP amplitude decreased with speed specifically in the midline cluster. This reduction in SSVEP amplitude could in principle reflect a global withdrawal of attention from visual input. Evidence against this interpretation comes from Andersen and Müller (2010) who found that reaction times in a detection task were predicted by the difference between attended and unattended SSVEP responses, but not by their sum. Therefore, inferring the strength of attentional selection from the overall weakening of SSVEPs affecting both targets and distractors is not well justified. Absolute SSVEP amplitudes are more likely linked to low-level stimulus properties (Di Russo et al., 2001; Walter et al., 2012). For example, Andersen et al. (2012) demonstrated that SSVEP amplitudes increase linearly with stimulus contrast for both attended and unattended stimuli, with attentional selection contributing an additional modulation on top. Amplitude changes that affect both targets and distractors are more likely to reflect changes in sensory input rather than changes in attentional allocation.

A more parsimonious explanation appeals to differences in receptive field size across the visual hierarchy (Smith et al., 2001). Midline electrodes likely reflect activity from earlier visual areas (V1–V3), which have smaller receptive fields than higher-order areas such as V4 or MT represented by lateral electrode clusters. At faster speeds, objects transit more rapidly through these smaller receptive fields, spending less time within any single receptive field and thereby producing weaker SSVEPs. In contrast, the larger receptive fields of higher visual areas may be better able to maintain continuous representation of fast-moving objects, resulting in speed-invariant attentional modulation in lateral clusters.

One alternative explanation for the speed invariance of attention in this study is that participants could, knowingly or unknowingly, select targets based on their common flicker frequency rather than the cue. To rule out this possibility, we carried out a separate behavioural control experiment with 17 different participants. This experiment used the same multiple object tracking paradigm as the main study but manipulated whether targets and distractors flickered at the same frequency or at two different frequencies. If participants were able to utilize flicker frequency as a cue for tracking, performance should be better when targets and distractors flicker at different frequencies. Participants tracked four targets amongst eight total objects moving at one of three speeds, identical to Experiment 1. In the critical manipulation, objects either 1) all flickered at 10 Hz, 2) all flickered at 12 Hz, 3) targets flickered at 10 Hz whilst distractors flickered at 12 Hz, or 4) vice versa. For analysis, these conditions were collapsed into two levels: frequency-same (Conditions 1–2) and frequency-different (Conditions 3–4). While tracking accuracy was lower at higher speeds, *F*_(2,32)_ = 29.99, *p* < 10^−7^, η^2^ = 0.28, it did not depend on whether all targets were flickering at the same frequency or not: Frequency: *F*_(1,16)_ = 2.89, *p* =.109, η^2^ = 0.04; Speed × Frequency interaction: *F*_(2,32)_ = 1.96, *p* =.157, η^2^ = 0.007. This indicates that participants were unable to use differences in flicker frequency as a cue for attentional selection, consistent with previous studies (Müller et al., 2006; Störmer et al., 2013).

## Experiment 2: Total number of objects

Having established that attentional selection of tracked targets remains robust across different speeds, we next examined whether increasing the total number of objects in the display dilutes attention distributed among a fixed number of tracked targets. This study is similar to Experiment 1, but instead of tracking targets at different speeds, participants tracked four targets among eight, 12, or 16 objects. Other spatial parameters of stimulation such as object sizes or total area remained unchanged. According to the flexible resources account, increased number of close encounters between targets and distractors depletes attention through higher demands on spatial resolution (Alvarez & Franconeri, 2007; Iordanescu et al., 2009). If this was the case, we expect to see weakened attentional selection with increased number of distractors.

### Method

#### Participants

Forty-eight University of Aberdeen students were recruited to participate in the study (10 men, 37 women, one nonbinary; two left-handed; *M*_age_ = 21.38, *SD*_age_ = 6.46). Five of them participated in the previous study. There were two reasons for the increased sample size compared with Experiment 1. First, we wanted to increase statistical power to detect any possible small interactions with attention. Second, we planned an analysis of individual differences whose purpose is orthogonal to the present paper, and which will be published separately. All participants reported normal or corrected-to-normal visual acuity and normal colour vision. The study received ethical approval from the Psychology Ethics Committee at the University of Aberdeen (No. 4412909).

Data from seven participants were excluded using the same exclusion rules as in Experiment 1. The final sample consisted of 41 participants.

#### Stimuli and procedure

**Fig. 3:**
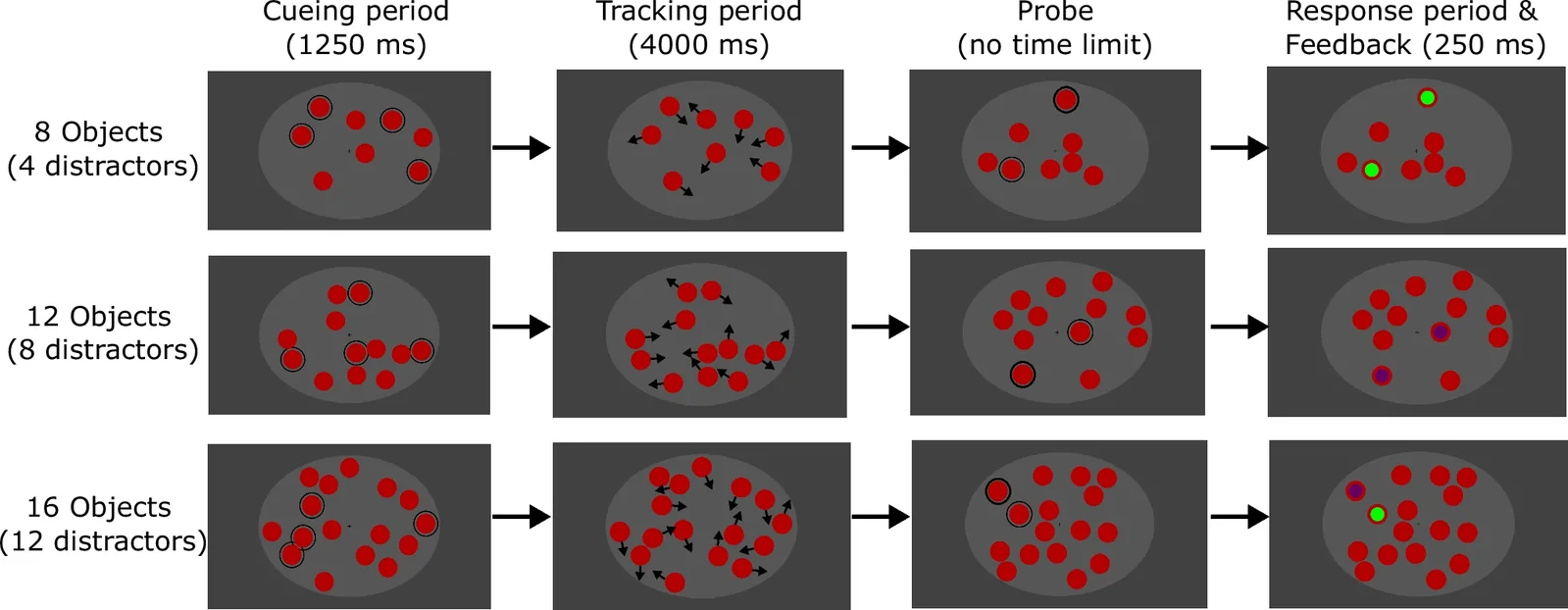
Illustration of the trial sequence in Experiment 2. Probes were presented and responses collected consecutively. Feedback displayed for both correct responses (top), both incorrect responses (middle), and one correct response (bottom). Black arrows during tracking period indicate motion directions and were not displayed. (Colour figure online)

Experiment 2 employed the same multiple object tracking paradigm but manipulated the total number of stimuli rather than their speed (Fig. 3). Participants tracked four targets amongst either eight, 12, or 16 total objects, all moving at a constant speed of 5.7 dva/s. The speed was determined during the piloting stage to avoid ceiling effects in behavioural performance. To alleviate spatial constraints within the fixed viewing area, the discs and the invisible surrounds was slightly reduced in size (3.8 dva diameter disks with additional 0.8 dva “bumpers”). The full report procedure yielded equal guessing chance across speed conditions in Experiment 1; however, this would not have been the case with respect to object number conditions in Experiment 2 as the proportion of targets in the display is systematically confounded with the total number of objects. Therefore, the response procedure was changed as follows. After each trial, two discs were consecutively probed, with targets and distractors probed with 50% probability. Participants were asked to report by key press whether each of the probed disks was a target or a distractor. This ensured comparable chance performance levels across conditions and experiments, thereby facilitating comparisons.

Irrespective of condition, throughout the trial four discs flickered at 10.9 Hz and four discs flickered at 13.3 Hz. In conditions where more than eight discs were presented, the remaining discs flickered at 12 Hz. To-be-tracked discs always included all four 10.9 Hz or all four 13.3 Hz discs allowing us to compare attentional enhancement of targets relative to distractors regardless of the total number of objects in the set. There were 336 trials in total with 56 trials per condition (three object number conditions for each of the two target stimulation frequencies).

After the EEG session, participants also completed the Adult Self-Report ADHD Scale (Kessler et al., 2005) and the Mind Excessively Wandering Scale (Mowlem et al., 2019), the results from which are a part of a separate project not reported in this manuscript.

#### Data analysis

Behavioural and EEG data analysis followed the same processing and analysis pipeline as in Experiment 1. Only trials with correct behavioural responses (to both probed stimuli) were included in the analysis. After removal of EEG artifacts and epochs preceding incorrect responses, the average trial rejection rate was 27.8% (±16.0%) with the 3.83 (±1.24) channels interpolated. Rescaled SSVEP amplitudes were analysed by means of repeated measures ANOVA with factors Attention (attended vs unattended) and Number of objects (eight, 12, and 16).

### Results

Tracking accuracy decreased in line with the number of stimuli on the screen, *F*_(2,80)_ = 63.23, *p* < 10^−16^, η^2^ = 0.06. Pairwise comparisons confirmed that tracking accuracy decreased when the total number of objects increased from eight (*M* = 0.86, *SD* = 0.05) to 12 (*M* = 0.81, *SD* = 0.06), *t*_(40)_ = 7.59, *p* < 10^−8^, *d* = 0.84, and further decreased from 12 to 16 objects (*M* = 0.77, *SD* = 0.07), *t*_(40)_ = 5.48, *p* < 10^−5^, *d* = 0.60.

#### SSVEP amplitudes

**Fig. 4:**
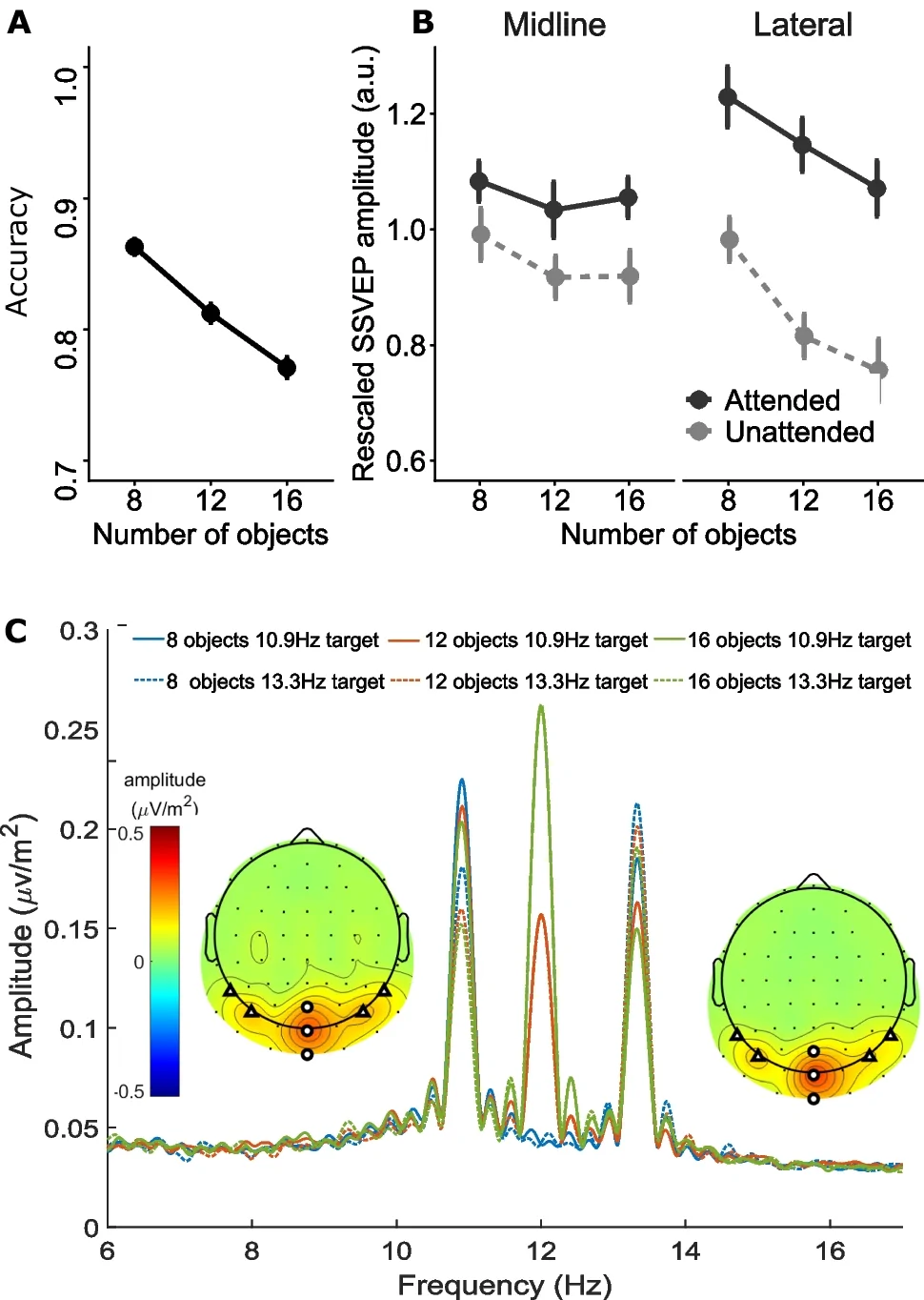
Results of Experiment 1. **A** Mean accuracy rates for three density conditions. **B** Rescaled grand mean SSVEP amplitudes and midline and lateral electrode clusters. **C** Grand-averaged amplitude spectrum of both electrode clusters combined, obtained by Fourier transformation and zero-padded to 16,384 points. The 12-Hz peak does not appear in eight object conditions as no objects flickered at 10 Hz. Inset: Grand mean scalp current density (SCD) maps of SSVEP amplitudes at 10.9 Hz (left) and 13.3 Hz (right) averaged across conditions. Circles on topographical maps indicate midline cluster electrodes, triangles indicate lateral cluster electrodes. Error bars denote within-subject 95% confidence intervals (Morey, 2008). (Colour figure online)

In the midline cluster of electrodes (Fig. 4B) SSVEP amplitudes were significantly higher for attended (*M* = 1.06, *SD* = 0.02) compared with unattended (*M* = 0.94, *SD* = 0.04) objects, *F*_(1,40)_ = 21.55, *p* < 10^−4^, η^2^ = 0.18 BF₁₀ = 6.91×10⁸. SSVEP amplitudes were significantly modulated by the total number of objects (eight: *M* = 1.04, *SD* = 0.07; 12: *M* = 0.98, *SD* = 0.08; 16: *M* = 0.99, *SD* = 0.10), *F*_(2,80)_ = 5.24, *p* =.007, η^2^ = 0.046, BF₁₀ = 3.3. Crucially, there was no significant interaction between the number of objects and attention, *F*_(2,80)_ = 0.97, *p* =.38, η^2^ = 0.005, BF₁₀ = 0.11, suggesting that the magnitude of attentional facilitation did not depend on the total number of objects in the set.

In the lateral cluster of electrodes, attention also enhanced SSVEP amplitudes (Attended: *M* = 1.15, *SD* = 0.08; Unattended: *M* = 0.85, *SD* = 0.12), *F*_(1,40)_ = 107.32, *p* < 10^−12^, η^2^ = 0.54, BF₁₀ = 1.32×10^31^. Amplitudes in both attended and unattended conditions decreased with the total number of objects (eight: *M* = 1.11, *SD* = 0.17; 12: *M* = 0.98, *SD* = 0.23, 16: *M* = 0.91, *SD* = 0.22), *F*_(2,80)_ = 42.82, *p* < 10^−9^, η^2^ = 0.25, BF₁₀ = 3607341. There was no interaction between the two factors, *F*_(2,80)_ = 2.63, *p* =.08, η^2^ = 0.017, BF₁₀ = 0.40.

Following the same analysis logic as the first experiment, to assess whether attention effects varied across conditions in the presence of the main effect of object number, we computed AMI for each condition separately. Contrary to the prediction of attention weakening, in the lateral cluster AMIs increased slightly with increasing number of objects (eight objects: *M* = 0.11, *SD* = 0.09; 12 objects: *M* = 0.17, *SD* = 0.08; 16 objects: *M* = 0.18, *SD* = 0.09), *F*_(2,80)_ = 5.81, *p* =.004, η^2^ = 0.05. AMIs in the midline cluster were not modulated by the number of objects, *F*_(2,80)_ = 1.09, *p* =.342, η^2^ = 0.009.

### Discussion

Increasing the number of objects in the display did reduce tracking performance as expected (Bettencourt & Somers, 2009; Drew et al., 2013; Feria, 2012), although this effect was less pronounced than the performance decrements observed with speed manipulations in Experiment 1 (see Figs. 2A & 3A), and in our previous work manipulating the number of targets to be tracked (Adamian & Andersen, 2022). This was not accompanied by a corresponding drop in attentional modulation of SSVEPs, which even exhibited a slight increase with increasing object numbers in the lateral electrode cluster. Therefore, increased tracking difficulty with more objects was not mediated by less effective attentional allocation.

The reduction in overall SSVEP amplitudes with increasing object number can be understood through the biased competition model of attention (Desimone & Duncan, 1995) according to which simultaneously presented stimuli activate overlapping neural populations and compete for cortical representation. This competition is affected by bottom-up (stimulus) and top-down (attentional) factors, biasing neural responses in favour of attended stimuli therefore allocating limited cortical resources to task-relevant objects (Luck et al., 1997; Reynolds & Chelazzi, 2004; Reynolds & Heeger, 2009). Critically, competition for neuronal representation is strongest when stimuli fall into shared receptive fields and attention affects neuronal firing rates most when this is the case (Moran & Desimone, 1985). As more objects are added to the display, they act in competitive suppression, reducing neural response to any individual object (Kastner et al., 1998, 2001). This explains why the lateral cluster—reflecting downstream visual areas with larger receptive fields—exhibits a more pronounced drop in SSVEP amplitudes with increasing object numbers and stronger attentional modulation than the midline cluster. More objects fall into its larger receptive fields, intensifying competition. Note that according to biased competition, the intensified competition for neural representation from adding more objects may lead to larger attentional modulation of visual processing, even if the attentional top-down signals themselves remain unchanged (however, Andersen et al., 2012, and Keitel et al., 2013, reported independent effects of competition and attention on SSVEP amplitudes).

Attention might facilitate tracking by reducing crowding between targets and distractors. Cueing spatial attention can decrease crowding effects (Yeshurun & Rashal, 2010). Some authors have suggested that crowding arises due to a limitation of precision of attention allocation (Intriligator & Cavanagh, 2001). If crowding was a limiting factor and this interpretation of crowding was true, we would expect attentional selectivity—the relative enhancement of targets over distractors—to be reduced in the condition with more distractors. However, this is not what we observed.

Consistent with Experiment 1, the dissociation between declining behavioural performance and maintained or enhanced attentional modulation demonstrates that tracking failures with increased object speed and number are not mediated by less effective attentional allocation. Similarly to limitations on tracking faster objects, spatial interference increases the probability of tracking errors even though the strength of attentional modulation is maintained.

### Template-based source localization of attention effects

The analyses carried out for Experiments 1 and 2 relied on electrode selection based on SSVEP amplitudes at the scalp level. Previous studies from our lab applying surface current density (SCD) transformation to SSVEP data have repeatedly identified independent electrode clusters whose activity was differentially modulated by selective attention (Adamian & Andersen, 2022; Adamian et al., 2020; Andersen et al., 2012). In the current study, our experimental manipulations also affected the two clusters in distinct ways: Midline cluster amplitudes were decreased with increasing object speed, whilst lateral cluster amplitudes were decreased with increasing object density. However, this method of electrode selection does not reveal the specific neural sources underlying these scalp-recorded signals. While we have anatomic grounds to assume that midline electrodes primarily reflect V1–V3 activity and lateral electrodes reflect activity in MT/V5/LOC, source localization can provide a more granular view of the cortical origins of attention effects.

To source localize attention effects, we applied a novel approach that uses fMRI-defined templates which specify how activity of different visual cortical areas appear on the scalp and therefore, in EEG data (Poncet & Ales, 2023). Templates created by Poncet and Ales (2023) are based on MRI and fMRI data of 50 participants and were generated by identifying retinotopic visual areas with fMRI, then simulating the expected EEG scalp distribution for each area. Templates are then fit to the observed EEG data using regularized regression to determine the contribution of each brain source to the recorded scalp activity. The templates and the accompanying EEG Source Template Matching toolbox are openly available.

### Method

Source localization was performed using EEG templates corresponding to average scalp activity for 18 visual cortical areas created by Poncet and Ales (2023). EEG data were referenced to the average. Complex Fourier coefficients were averaged across participants and submitted to the template matching algorithm, resulting in estimated activity for each condition and each region of interest (ROI). Since the template matching method uses group-level data, statistical tests were based on bootstrap resampling with replacement (1,000 iterations).

For each bootstrap sample, AMIs were calculated separately for each ROI, then averaged across bilateral ROIs and across the two stimulation frequencies to reduce multiple comparisons. Additionally, AMIs were averaged between ventral and dorsal V2 and V3 to account for full-field stimulation, resulting in seven ROIs in total: V1, V2, V3, V4, V3A, LOC, and hMT+. To identify cortical ROIs significantly modulated by attention, we performed two-sided bootstrap *t* tests of each condition-average AMI against zero.

### Results

**Fig. 5:**
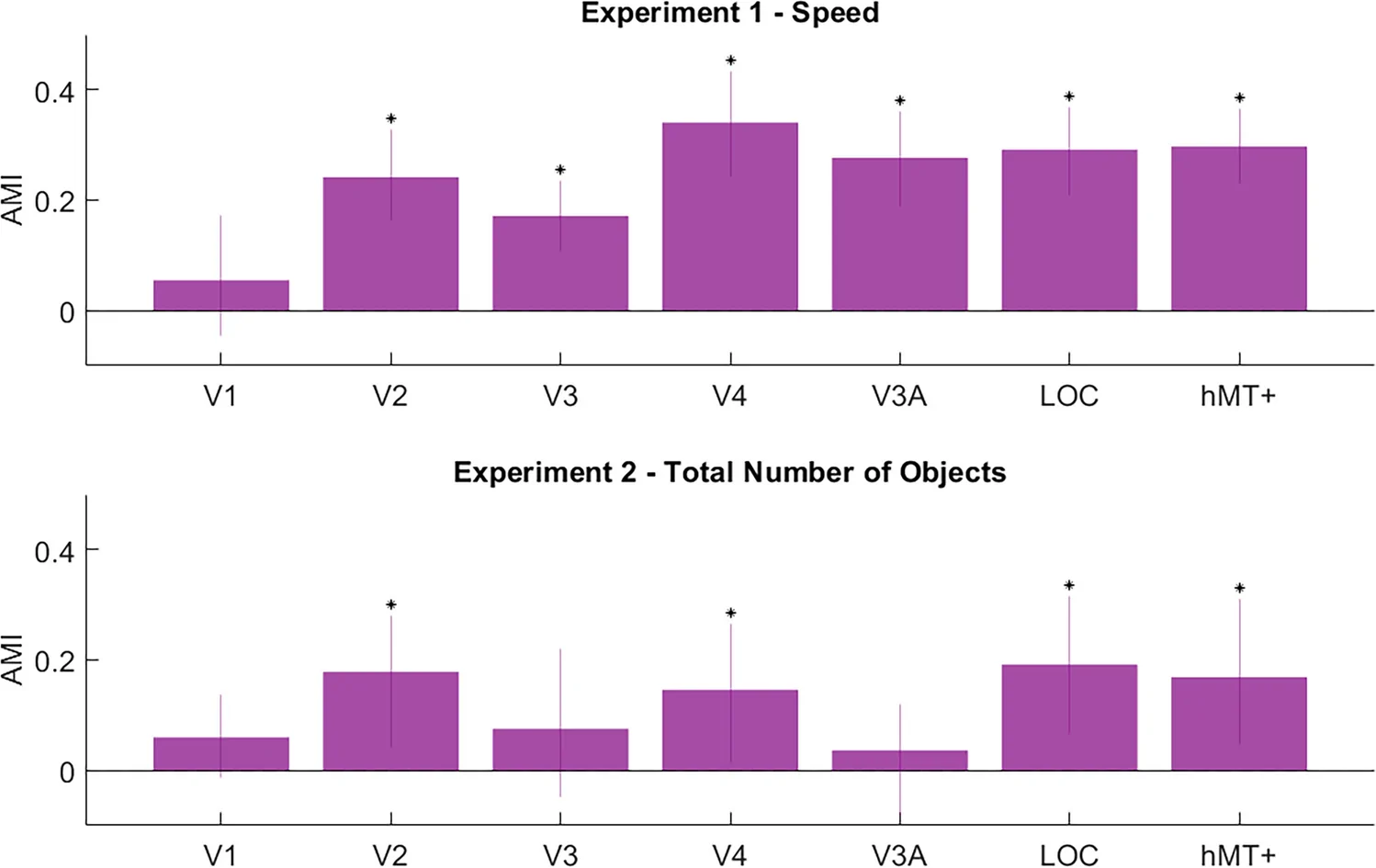
Results of the source localisation analysis for Experiment 1 (top) and Experiment 2 (bottom). Error bars are 95% CIs. Stars indicate statistically significant AMIs

In Experiment 1 where object speed was manipulated, six out of seven visual ROIs showed significant modulation of SSVEP amplitudes by attention (see Fig. 5; *p* values <.001), with the exception of area V1 *(p* =.26). In Experiment 2, where total number of objects varied by condition, significant attentional modulation was observed in V2 (*p* =.02), V4 (*p *=.03), LOC (*p* =.01), and hMT+ (*p* =.01). Again, SSVEP signal originating in V1 was not modulated by attention (*p *=.08). The absence of significant attentional modulation in V1 may reflect its small receptive fields, which cannot integrate SSVEPs as tagged objects move through space. This likely results in weak or unstable SSVEP responses in V1, leaving little signal to be enhanced by attention. While V1 along with MT is an established major source of SSVEP (Di Russo et al., 2006), it is unsurprising that the use of moving stimuli would shift the balance of SSVEP amplification towards extrastriate visual areas.

Areas V3 and V3A showed attentional modulation only in the speed manipulation experiment. While speculative, this could reflect enhanced motion processing associated with successful tracking of faster moving objects (Tootell et al., 1997).

These results are in broad agreement with previous studies identifying peak modulation of SSVEP amplitudes in early visual cortex (V1–V3) and MT/LOC (Adamian et al., 2020; Störmer et al., 2013), validating template matching as an effective method for localizing attention effects in SSVEP experiments. Compared with phase-based electrode clustering, this approach provides finer anatomical resolution by separating signals from adjacent visual areas that could otherwise be conflated in electrode-level analyses.

## General discussion

In two experiments, we investigated if object speed and number of distractors modulate attentional allocation during multiple object tracking in the same manner as top-down task demands. In Experiment 1, we manipulated object speed whilst holding the number of targets and distractors constant. Behaviourally, tracking performance declined as speed increased, replicating well-established findings (Drew et al., 2013; Feria, 2013). However, SSVEPs revealed that attentional modulation of tracked targets relative to unattended distractors did not vary with object speed during successful tracking trials. A behavioural control experiment further confirmed that the different flicker frequencies between targets and distractors did not benefit tracking performance. In Experiment 2, we manipulated the total number of objects in the display whilst keeping the number of targets constant. Again, tracking performance declined as more objects were added, yet attentional modulation either remained constant (midline electrode cluster) or slightly strengthened (lateral electrode cluster) with increased object number. These findings contrast sharply with the effects of tracking more targets: When the number of tracked targets increases, attentional modulation of each individual target systematically weakens (Adamian & Andersen, 2022). Together, these results demonstrate that bottom-up display characteristics and top-down task demands affect tracking in fundamentally different ways. Although these manipulations may trade-off behaviourally (Alvarez & Franconeri, 2007), they have qualitatively different effects on visual cortical processing of targets and distractors. Consequently, even if conditions in which different numbers of targets tracked are matched in tracking performance by covarying physical stimulus parameters, this may result in perceptual or behavioural differences in other measures than tracking performance.

This dynamic can be explained using a classic metaphor of sticky references (Pylyshyn, 1989), where attention is thought of as “glue” binding targets to their representations in visual cortex. When tracking more targets, the same amount of glue must be spread more thinly, and each target receives less attentional facilitation (Adamian & Andersen, 2022). However, with increased bottom-up demands such as speed or number of targets, the amount of glue stays the same while external forces pulling targets away from their representations (e.g., close encounters) become stronger and/or more frequent. Metaphorically speaking, the glue is subjected to stress until it can no longer hold. The amount or strength of glue (attentional allocation) remains constant, but it becomes insufficient to maintain stable tracking against heightened interference, resulting in more errors. In this view, there is no gradual weakening of attention in physical stimulus conditions that are more challenging for tracking. However, attentional selection may break down for individual objects when discrete tracking errors occur, such as the loss of a target object (“dropping”) or a close encounter between objects leading to erroneous tracking of a distractor object instead (“swapping”; Drew et al., 2013). For this reason, SSVEP amplitudes during tracking predict behavioural performance at the end of the trial (Adamian & Andersen, 2022; Störmer et al., 2013).

The pattern of results supports target enhancement rather than distractor suppression as the primary attentional mechanism subserving object tracking (Bettencourt & Somers, 2009). If tracking relied on active suppression of distractors, one would predict that attentional modulation should decrease with increasing numbers of distractors. However, both the current findings and our previous work (Adamian & Andersen, 2022) demonstrate the opposite pattern: attentional modulation is sensitive to the number of tracked targets but remains stable across variations in distractor number. This dissociation strongly suggests that the visual system prioritises enhancing target representations rather than suppressing distractor processing during multiple object tracking.

Although neither experimental manipulation affected the magnitude of target enhancement over distractors, both affected visual processing as indexed by the SSVEPs, specifically in the midline cluster in Experiment 1 and in the lateral cluster in Experiment 2. These influences are more readily explained by low-level sensory changes than by a withdrawal of attention, which would require attention to be removed equally from targets and distractors.^1^ Moreover, reaction times have been previously linked to the relative, but not the total, SSVEP amplitudes elicited by targets and distractors (Andersen & Muller, 2010), leaving little support for an attentional account of the overall amplitude reduction.

The dissociation between top-down attentional control and bottom-up task demands is consistent with prior evidence. Neuroimaging studies have revealed functionally distinct neural networks for general task engagement versus tracking specific numbers of targets (Culham et al., 2001; Felßberg et al., 2025; Mäki-Marttunen et al., 2020). Behavioural work has similarly suggested that spatial interference imposes limits on tracking that are separable from attentional capacity limits (Meyerhoff et al., 2016; Vul et al., 2009). The present findings add to this evidence by demonstrating that manipulations affecting bottom-up processing demands such as object speed or distractor number are orthogonal to top-down attentional selection during successful tracking.

To conclude, while tracking is accomplished through multifocal attention, it is not less effective allocation of selective attention that limits tracking capacity under high-speed or high-distractor-number conditions. Attention is allocated just as effectively but does not afford the same level of tracking performance under these more challenging conditions.

## Data Availability

Participant-level data (EEG and behavioural) and aggregated data with analysis code is available: https://osf.io/ypgfs

## Footnotes

1 Note also that a change affecting attended and unattended stimuli equally cannot readily be attributed to selective attention, as such a change is in itself nonselective.

## Code availability

Code producing data visualization and statistical analyses is available: https://osf.io/ypgfs

## Declarations

### Open practices statement

Participant-level data and analysis code for all experiments are available online (https://osf.io/ypgfs/), and Experiment 1 was preregistered (https://osf.io/pxh25/).

## References

[CR1] Adamian, N., & Andersen, S. K. (2022). Attentional enhancement of tracked stimuli in early visual cortex has limited capacity. *Journal of Neuroscience,**42*, 8836–8844. 10.1523/JNEUROSCI.0605-22.2022 PMC9671574 36202616

[CR2] Adamian, N., & Andersen, S. K. (2024). Attentional modulation in early visual cortex: A focused reanalysis of steady-state visual evoked potential studies. *Journal of Cognitive Neuroscience,**36*(1), 46–70. 10.1162/jocn_a_02070 37847846

[CR3] Adamian, N., Andersen, S. K., & Hillyard, S. A. (2020). Parallel attentional facilitation of features and objects in early visual cortex. *Psychophysiology,**57*(3), Article e13498. 10.1111/psyp.13498 PMC7027440 31691314

[CR4] Alvarez, G. A., & Franconeri, S. L. (2007). How many objects can you track?: Evidence for a resource-limited attentive tracking mechanism. *Journal of Vision,**7*(13), Article 14. 10.1167/7.13.14 17997642

[CR5] Andersen, S. K., & Muller, M. M. (2010). Behavioral performance follows the time course of neural facilitation and suppression during cued shifts of feature-selective attention. *Proceedings of the National Academy of Sciences of the United States of America,**107*(31), 13878–13882. 10.1073/pnas.1002436107 PMC2922290 20643918

[CR6] Andersen, S. K., Muller, M. M., & Martinovic, J. (2012). Bottom-up biases in feature-selective attention. *Journal of Neuroscience,**32*(47), 16953–16958. 10.1523/JNEUROSCI.1767-12.2012 PMC6621795 23175846

[CR7] Bettencourt, K. C., & Somers, D. C. (2009). Effects of target enhancement and distractor suppression on multiple object tracking capacity. *Journal of Vision,**9*(7), 9. 10.1167/9.7.9 19761324

[CR8] Chen, J., McManus, M., Valsecchi, M., Harris, L. R., & Gegenfurtner, K. R. (2019). Steady-state visually evoked potentials reveal partial size constancy in early visual cortex. *Journal of Vision*, *19*(6), Article 8. 10.1167/19.6.8 31185096

[CR9] Culham, J. C., Cavanagh, P., & Kanwisher, N. G. (2001). Attention response functions: Characterizing brain areas using fMRI activation during parametric variations of attentional load. *Neuron,**32*(4), 737–745. 10.1016/s0896-6273(01)00499-8 11719212

[CR10] Delorme, A., & Makeig, S. (2004). EEGLAB: An open source toolbox for analysis of single-trial EEG dynamics including independent component analysis. *Journal of Neuroscience Methods,**134*(1), 9–21. 10.1016/j.jneumeth.2003.10.009 15102499

[CR11] Desimone, R., & Duncan, J. (1995). Neural mechanisms of selective visual attention. *Annual Review of Neuroscience,**18*(1), 193–222. 10.1146/annurev.ne.18.030195.001205 7605061

[CR12] Di Russo, F., Pitzalis, S., Aprile, T., Spitoni, G., Patria, F., Stella, A., Spinelli, D., & Hillyard, S. A. (2006). Spatiotemporal analysis of the cortical sources of the steady-state visual evoked potential. *Human Brain Mapping,**28*(4), 323–334. 10.1002/hbm.20276 PMC6871301 16779799

[CR13] Di Russo, F., Spinelli, D., & Morrone, M. C. (2001). Automatic gain control contrast mechanisms are modulated by attention in humans: Evidence from visual evoked potentials. *Vision Research,**41*(19), 2435–2447. 10.1016/S0042-6989(01)00134-1 11483175

[CR14] Drew, T., Horowitz, T. S., & Vogel, E. K. (2013). Swapping or dropping? Electrophysiological measures of difficulty during multiple object tracking. *Cognition,**126*(2), 213–223. 10.1016/j.cognition.2012.10.003 PMC3529852 23141025

[CR15] Felßberg, A.-M., Schönemann, N., Hopf, J.-M., Schoenfeld, M. A., & Merkel, C. (2025). Cortical network modulations associated with prolonged training of the multiple object-tracking task. *Imaging Neuroscience,**3*, Article imag_a_00577. 10.1162/imag_a_00577 PMC12319924 40800779

[CR16] Feria, C. S. (2012). The effects of distractors in multiple object tracking are modulated by the similarity of distractor and target features. *Perception,**41*(3), 287–304. 10.1068/p7053 22808583

[CR17] Feria, C. S. (2013). Speed has an effect on multiple-object tracking independently of the number of close encounters between targets and distractors. *Attention, Perception, & Psychophysics,**75*(1), 53–67. 10.3758/s13414-012-0369-x 22972631

[CR18] Franconeri, S. L., Jonathan, S. V., & Scimeca, J. M. (2010). Tracking multiple objects is limited only by object spacing, not by speed, time, or capacity. *Psychological Science,**21*(7), 920–925. 10.1177/0956797610373935 20534781

[CR19] Franconeri, S. L., Lin, J. Y., Enns, J. T., Pylyshyn, Z. W., & Fisher, B. (2008). Evidence against a speed limit in multiple-object tracking. *Psychonomic Bulletin & Review,**15*(4), 802–808. 10.3758/PBR.15.4.802 18792507

[CR20] Hedjar, L., Martinovic, J., Andersen, S. K., & Shapiro, A. G. (2025). Separation of luminance and contrast modulation in steady-state visual evoked potentials. *Vision Research,**230*, Article 108567. 10.1016/j.visres.2025.108567 40054086

[CR21] Holcombe, A. (2023). Attending to moving objects. *Elements in Perception*. 10.1017/9781009003414

[CR22] Holcombe, A., & Chen, W.-Y. (2012). Exhausting attentional tracking resources with a single fast-moving object. *Cognition,**123*(2), 218–228. 10.1016/j.cognition.2011.10.003 22055340

[CR23] Intriligator, J., & Cavanagh, P. (2001). The spatial resolution of visual attention. *Cognitive Psychology,**43*(3), 171–216. 10.1006/cogp.2001.0755 11689021

[CR24] Iordanescu, L., Grabowecky, M., & Suzuki, S. (2009). Demand-based dynamic distribution of attention and monitoring of velocities during multiple-object tracking. *Journal of Vision,**9*(4), 1. 10.1167/9.4.1 PMC2756460 19757910

[CR25] Junghöfer, M., Elbert, T., Tucker, D. M., & Rockstroh, B. (2000). Statistical control of artifacts in dense array EEG/MEG studies. *Psychophysiology, 37*(4), 523–532 10934911

[CR26] Kaneko, S., Kuriki, I., & Andersen, S. K. (2020). Steady-state visual evoked potentials elicited from early visual cortex reflect both perceptual color space and cone-opponent mechanisms. *Cerebral Cortex Communications,**1*(1), Article tgaa059. 10.1093/texcom/tgaa059 PMC8152915 34296122

[CR27] Kastner, S., De Weerd, P., Desimone, R., & Ungerleider, L. G. (1998). Mechanisms of directed attention in the human extrastriate cortex as revealed by functional MRI. *Science,**282*(5386), 108–111. 10.1126/science.282.5386.108 9756472

[CR28] Kastner, S., De Weerd, P., Pinsk, M. A., Elizondo, M. I., Desimone, R., & Ungerleider, L. G. (2001). Modulation of sensory suppression: Implications for receptive field sizes in the human visual cortex. *Journal of Neurophysiology,**86*(3), 1398–1411. 10.1152/jn.2001.86.3.1398 11535686

[CR29] Keitel, C., Andersen, S. K., Quigley, C., & Muller, M. M. (2013). Independent effects of attentional gain control and competitive interactions on visual stimulus processing. *Cerebral Cortex,**23*(4), 940–946. 10.1093/cercor/bhs084 22510530

[CR30] Kessler, R. C., Adler, L., Ames, M., Demler, O., Faraone, S., Hiripi, E., Demler, O. L. G. A., Hiripi, E. V. A., Howes, M. A. R. Y. J., Jin, R. O. B. E. R. T., Secnik, K. R. I. S. T. I. N. A., Spencer, T. H. O. M. A. S., Ustun, T. B. E. D. I. R. H. A. N., & Walters, E. E. (2005). The World Health Organization adult ADHD self-report scale (ASRS): A short screening scale for use in the general population. *Psychological Medicine,**35*(2), 245–256. 10.1017/S0033291704002892 15841682

[CR31] Kleiner, M., Brainard, D., & Pelli, D. (2007). *What’s new in Psychtoolbox-3?* https://pure.mpg.de/rest/items/item_1790332/component/file_3136265/content

[CR32] Liu, G., Austen, E. L., Booth, K. S., Fisher, B. D., Argue, R., Rempel, M. I., & Enns, J. T. (2005). Multiple-object tracking is based on scene, not retinal, coordinates. *Journal of Experimental Psychology: Human Perception and Performance,**31*(2), 235–247. 10.1037/0096-1523.31.2.235 15826227

[CR33] Luck, S. J., Chelazzi, L., Hillyard, S. A., & Desimone, R. (1997). Neural mechanisms of spatial selective attention in areas V1, V2, and V4 of macaque visual cortex. *Journal of Neurophysiology,**77*(1), 24–42. 10.1152/jn.1997.77.1.24 9120566

[CR34] Mäki-Marttunen, V., Hagen, T., Laeng, B., & Espeseth, T. (2020). Distinct neural mechanisms meet challenges in dynamic visual attention due to either load or object spacing. *Journal of Cognitive Neuroscience,**32*(1), 65–84. 10.1162/jocn_a_01469 31560270

[CR35] Martinovic, J., Wuerger, S. M., Hillyard, S. A., Müller, M. M., & Andersen, S. K. (2018). Neural mechanisms of divided feature-selective attention to colour. *NeuroImage,**181*, 670–682. 10.1016/j.neuroimage.2018.07.033 30048748

[CR36] Meyerhoff, H. S., Papenmeier, F., & Huff, M. (2017). Studying visual attention using the multiple object tracking paradigm: A tutorial review. *Attention, Perception, & Psychophysics,**79*(5), 1255–1274. 10.3758/s13414-017-1338-1 28584953

[CR37] Meyerhoff, H. S., Papenmeier, F., Jahn, G., & Huff, M. (2016). Not FLEXible enough: Exploring the temporal dynamics of attentional reallocations with the multiple object tracking paradigm. *Journal of Experimental Psychology: Human Perception and Performance,**42*(6), 776–787. 10.1037/xhp0000187 26689311

[CR38] Moran, J., & Desimone, R. (1985). Selective attention gates visual processing in the extrastriate cortex. *Science,**229*(4715), 782–784. 10.1126/science.4023713 4023713

[CR39] Morey, R. D. (2008). Confidence intervals from normalized data: A correction to Cousineau (2005). *Tutorials in Quantitative Methods for Psychology,**4*(2), 61–64. 10.20982/tqmp.04.2.p061

[CR40] Morgan, S. T., Hansen, J. C., & Hillyard, S. A. (1996). Selective attention to stimulus location modulates the steady-state visual evoked potential. *Proceedings of the National Academy of Sciences,**93*(10), 4770–4774. 10.1073/pnas.93.10.4770 PMC39354 8643478

[CR41] Mowlem, F. D., Skirrow, C., Reid, P., Maltezos, S., Nijjar, S. K., Merwood, A., Barker, Edward, Cooper, Ruth, Kuntsi, Jonna, & Asherson, P. (2019). Validation of the mind excessively wandering scale and the relationship of mind wandering to impairment in adult ADHD. *Journal of Attention Disorders,**23*(6), 624–634. 10.1177/1087054716651927 PMC6429624 27255536

[CR42] Müller, M. M., Andersen, S., Trujillo, N. J., Valdés-Sosa, P., Malinowski, P., & Hillyard, S. A. (2006). Feature-selective attention enhances color signals in early visual areas of the human brain. *Proceedings of the National Academy of Sciences of the United States of America,**103*(38), 14250–14254. 10.1073/pnas.0606668103 PMC1599943 16956975

[CR43] Norcia, A. M., Appelbaum, L. G., Ales, J. M., Cottereau, B. R., & Rossion, B. (2015). The steady-state visual evoked potential in vision research: A review. *Journal of Vision,**15*(6), 4–4. 10.1167/15.6.4 PMC4581566 26024451

[CR44] Perrin, F., Pernier, J., Bertrand, O., & Echallier, J. F. (1989). Spherical splines for scalp potential and current density mapping. *Electroencephalography and Clinical Neurophysiology,**72*(2), 184–187. 10.1016/0013-4694(89)90180-6 2464490

[CR45] Poncet, M., & Ales, J. M. (2023). Estimating neural activity from visual areas using functionally defined EEG templates. *Human Brain Mapping,**44*(5), 1846–1861. 10.1002/hbm.26188 PMC9980892 36655286

[CR46] Pylyshyn, Z. (1989). The role of location indexes in spatial perception: A sketch of the FINST spatial-index model. *Cognition,**32*(1), 65–97. 10.1016/0010-0277(89)90014-0 2752706

[CR47] Pylyshyn, Z., & Storm, R. W. (1988). Tracking multiple independent targets: Evidence for a parallel tracking mechanism. *Spatial Vision,**3*(3), 179–197. 10.1163/156856888x00122 3153671

[CR48] Reynolds, J. H., & Chelazzi, L. (2004). Attentional modulation of visual processing. *Annual Review of Neuroscience,**27*, 611–647. 10.1146/annurev.neuro.26.041002.131039 15217345

[CR49] Reynolds, J. H., & Heeger, D. J. (2009). The normalization model of attention. *Neuron,**61*(2), 168–185. 10.1016/j.neuron.2009.01.002 PMC2752446 19186161

[CR50] Smith, A. T., Singh, K. D., Williams, A. L., & Greenlee, M. W. (2001). Estimating receptive field size from fMRI data in human striate and extrastriate visual cortex. *Cerebral Cortex,**11*(12), 1182–1190. 10.1093/cercor/11.12.1182 11709489

[CR51] Störmer, V. S., Winther, G. N., Li, S.-C., & Andersen, S. K. (2013). Sustained multifocal attentional enhancement of stimulus processing in early visual areas predicts tracking performance. *Journal of Neuroscience,**33*(12), 5346–5351. 10.1523/JNEUROSCI.4015-12.2013 PMC6704982 23516299

[CR52] Tombu, M., & Seiffert, A. E. (2011). Tracking planets and moons: Mechanisms of object tracking revealed with a new paradigm. *Attention, Perception, & Psychophysics,**73*(3), 738–750. 10.3758/s13414-010-0060-z PMC4150655 21264704

[CR53] Tootell, R. B., Mendola, J. D., Hadjikhani, N. K., Ledden, P. J., Liu, A. K., Reppas, J. B., Tootell, R. B. H., Sereno, M. I., & Dale, A. M. (1997). Functional analysis of V3A and related areas in human visual cortex. *The Journal of Neuroscience,**17*(18), 7060–7078. 10.1523/JNEUROSCI.17-18-07060.1997 PMC6573277 9278542

[CR54] Vul, E., Alvarez, G., Tenenbaum, J., & Black, M. (2009). Explaining human multiple object tracking as resource-constrained approximate inference in a dynamic probabilistic model. *Advances in Neural Information Processing Systems*, *22*. https://papers.nips.cc/paper_files/paper/2009/hash/d79aac075930c83c2f1e369a511148fe-Abstract.html

[CR55] Walter, S., Quigley, C., Andersen, S. K., & Mueller, M. M. (2012). Effects of overt and covert attention on the steady-state visual evoked potential. *Neuroscience Letters,**519*(1), 37–41. 10.1016/j.neulet.2012.05.011 22579858

[CR56] Yeshurun, Y., & Rashal, E. (2010). Precueing attention to the target location diminishes crowding and reduces the critical distance. *Journal of Vision,**10*(10), 16. 10.1167/10.10.16 20884481

